# Use of low-dose CT KUB: is it becoming the easy way out?

**DOI:** 10.1186/cc13275

**Published:** 2014-03-17

**Authors:** P Shah, D Wilson

**Affiliations:** 1 Homerton University Hospital, London, UK

## Introduction

In 1995, Smith and colleagues first proposed the use of low-dose CT KUB for the diagnosis of ureteric colic [1]. Since then, popularity of this imaging modality has increased due to a number of reasons: a sensitivity of 94 to 97%, a specificity of 96 to 99%, lack of intravenous contrast injection and speed of examination. The UK College of Emergency Medicine considers CT KUB as best practice for radiological investigation of renal colic. A review of the literature suggests that the true positive rate (number of patients diagnosed with an obstructing or symptom causing calculus) should be between 47.5 and 67% and alternative diagnoses should be confirmed in approximately 10% of patients imaged [2].

## Methods

All patients over the age of 18 years who had a CT KUB requested from the emergency department of the Homerton University Hospital, London over a period of 3 months, between May 2012 and July 2012, were identified. Individual case notes were examined and data were collected on an Excel spreadsheet. Data were analysed and results were divided into positive for renal colic (true positive), positive for other pathology (other significant diagnoses) and negative results.

## Results

A similar previous audit carried out in 2009 at the same hospital demonstrated a true positive rate of 48.5%. The 2012 audit examined the outcome of 124 consecutive scans and a true positive rate of 29% (*P *= 0.0006) was identified. Alternative diagnoses were 10% in 2009 compared with 17% in 2012 (*P *= 0.02).

## Conclusion

The true positive rate of CT KUB for renal colic has decreased significantly. We are getting more negative scan results and more scans diagnosing other significant pathology. The authors believe clinicians' thresholds for imaging may have decreased due to the apparent low-dose radiation of CT KUBs. Furthermore, there is a perceived ease of access to CT KUB imaging and hence this modality appears to be being used to identify other significant pathology.
